# Curcumin and metformin synergistically modulate peripheral and central immune mechanisms of pain

**DOI:** 10.1038/s41598-022-13647-7

**Published:** 2022-06-11

**Authors:** Peththa Wadu Dasuni Wasana, Hasriadi, Chawanphat Muangnoi, Opa Vajragupta, Pranee Rojsitthisak, Pornchai Rojsitthisak, Pasarapa Towiwat

**Affiliations:** 1 Pharmaceutical Sciences and Technology Program, Faculty of Pharmaceutical Sciences, Chulalongkorn University, Bangkok, 10330 Thailand; 2 Department of Pharmacology and Physiology, Faculty of Pharmaceutical Sciences, Chulalongkorn University, Bangkok, 10330 Thailand; 3 Institute of Nutrition, Mahidol University, Salaya, Nakhon Pathom, 73170 Thailand; 4 Molecular Probes for Imaging Research Network, Faculty of Pharmaceutical Sciences, Chulalongkorn University, Bangkok, 10330 Thailand; 5 Metallurgy and Materials Science Research Institute, Chulalongkorn University, Bangkok, 10330 Thailand; 6 Center of Excellence in Natural Products for Ageing and Chronic Diseases, Chulalongkorn University, Bangkok, 10330 Thailand; 7 Department of Food and Pharmaceutical Chemistry, Faculty of Pharmaceutical Sciences, Chulalongkorn University, Bangkok, 10330 Thailand

**Keywords:** Drug discovery, Pain

## Abstract

Metformin is a well-tolerated antidiabetic drug and has recently been repurposed for numerous diseases, including pain. However, a higher dose of metformin is required for effective analgesia, which can potentiate its dose-dependent gastrointestinal side effects. Curcumin is a natural polyphenol and has beneficial therapeutic effects on pain. Curcumin has been used as an analgesic adjuvant with several analgesic drugs, allowing synergistic antinociceptive effects. Nevertheless, whether curcumin can exert synergistic analgesia with metformin is still unknown. In the present study, the nature of curcumin-metformin anti-inflammatory interaction was evaluated in in vitro using lipopolysaccharide-induced RAW 264.7 macrophage and BV-2 microglia cells. In both macrophage and microglia, curcumin effectively potentiates the anti-inflammatory effects of metformin, indicating potential synergistic effects in both peripheral and central pathways of pain. The nature of the interaction between curcumin and metformin was further recapitulated using a mouse model of formalin-induced pain. Coadministration of curcumin and metformin at a 1:1 fixed ratio of their ED_50_ doses significantly reduced the dose required to produce a 50% effect compared to the theoretically required dose in phase II of the formalin test with a combination index value of 0.24. Besides, the synergistic interaction does not appear to involve severe CNS side effects indicated by no motor alterations, no alterations in short-term and long-term locomotive behaviors, and the general well-being of mice. Our findings suggest that curcumin exerts synergistic anti-inflammation with metformin with no potential CNS adverse effects.

## Introduction

Pain is defined as “an unpleasant sensory and emotional experience associated with, or resembling that associated with, actual or potential tissue damage”^1^. Though pain serves a protective function, the excessive triggering of nociceptors by inflammatory mediators leads to a devastating condition identified as inflammatory pain^2^. Pathophysiology of inflammatory pain is characterized by an excessive peripheral and central immune response, indicated by increased release of inflammatory mediators, including cytokines, chemokines, proteases, and growth factors^3^. The activation of peripheral and central immune cells contributes to enhanced pain transmission via peripheral and central sensitization, respectively^3,4^. Several treatment options are currently available to treat inflammatory pain, including non-steroidal anti-inflammatory drugs, steroids, and opioids. Yet, the side effects of current treatments, including gastrointestinal irritation, cardiovascular toxicity, nephrotoxicity, and dependence (opioids), continued to be an issue^5^. Given these issues, searching for alternative treatments for pain management is imperative.

**Figure 1:**
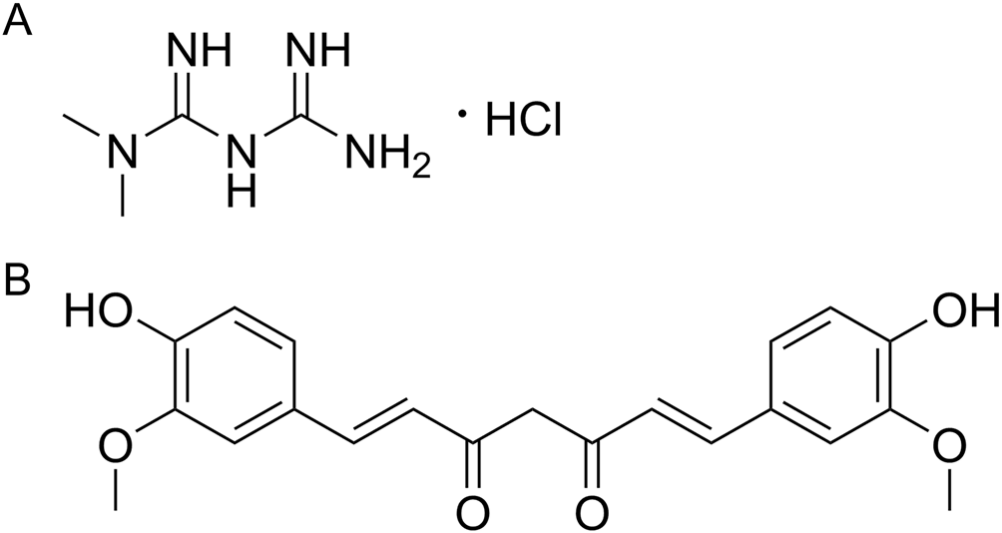
Chemical structures of metformin hydrochloride (**A**) and curcumin (**B**).

Metformin is a well-tolerated antidiabetic drug (Fig. 1A), and recently several pre-clinical and clinical studies have shown the potential analgesic effects of metformin^6–10^. Given that this drug is a disease-modifying agent in type II diabetes mellitus, it is possible that it has similar properties for reducing pain and its comorbidities^11^. Metformin has been shown to alleviate pain-like behaviors by activating the AMP-activated protein kinase (AMPK) and opioidergic pathways^6,12^. In rodent models of pain, metformin was found to inhibit peripheral and central inflammation, major contributing factors of pain progression, along with reflexive and non-reflexive pain behaviors^13,14^. However, a clinical study conducted on diabetic patients indicated the necessity of using a higher dose of metformin in humans to be a potential analgesic^15^. The use of a higher dose of metformin may increase the chance of experiencing side effects associated with metformin which include most commonly gastrointestinal disturbances: diarrhea and nausea, abdominal discomfort and indigestion, and hypoglycemia^16^. The undesirable gastrointestinal side effects of metformin were observed in ~ 25% of patients, where ~ 5% were unable to tolerate it^17^. The side effect of metformin also has been associated with lactic acidosis^18^. Hence, it is essential to lower the analgesic dose requirement of metformin, which can be achieved by applying several approaches, including the drug combination and nanoparticle approaches.

Curcumin ([1E,6E]-1,7-bis[4-hydroxy-3-methoxyphenyl]-1,6-heptadiene-3,5-dione) is a naturally occurring polyphenolic compound (Fig. 1B), first isolated from the herbaceous perennial plant, *Curcuma longa* (turmeric)*.* Curcumin has been used as a traditional herbal remedy for centuries throughout Asia due to its pleiotropic activities, including anti-inflammatory, antioxidant, and anticancer^19^. It is a well‐tolerated natural product causing no or minimal toxicity in short- and long-term use^20^. Consequently, it was declared “generally recognized as safe” by the US Food and Drug Administration (FDA)^21^. Moreover, the therapeutic effectiveness of curcumin in nociceptive, inflammatory, and neuropathic pain has been reported in numerous animal models and humans^22^. Considering its analgesic mechanisms of action, it effectively attenuates pain by modulating the neurotransmitters related to pain, suppressing the immune response, or blocking the transient receptor potential vanilloid type I (TRPV1) receptors, and modulating purinergic and chemokine receptors^23–27^. However, curcumin has low stability and poor water solubility and is quickly metabolized in the gastrointestinal tract and liver, despite its apparent benefits^27^.

In the last few years, research has shown that herb-drug combinations in pain management produce higher efficacy and lesser adverse effects than a single drug administration^28^. This could be due to their ability to target several sites of the pain pathway, which minimizes the effective doses of both compounds in the combination. Dual treatment with curcumin and metformin has been reported in diabetic mellitus^29^, diabetes-induced comorbidities^30^, nephrotoxicity^31^, hepatocellular carcinoma, pancreatic cancer cells, and breast cancer^32^, with the results suggesting synergistic effects^31^. Moreover, metformin and curcumin have different mechanisms of action in pain modulation, which indicates the potential for exerting greater analgesia when administered together. Hence, this study aims to evaluate the nature of the pharmacological interaction between curcumin and metformin in both the peripheral and central levels of pain transmission to identify their potential use in analgesia.

## Results and discussion

### Curcumin, in combination with metformin, synergistically inhibited the NO production of LPS-stimulated RAW 264.7 macrophage and BV-2 microglial cells

Macrophage and microglia, immune cells located in the peripheral and central nervous systems (PNS and CNS), respectively, play a major role in the pathogenesis of inflammatory pain via neuroimmune crosstalk. Immune cells in the PNS and CNS produce mediators (proinflammatory cytokines and chemokines) that modulate pain sensitivity. Nociceptor neurons, in turn, release neuropeptides and neurotransmitters that regulate the immune cell responses. Moreover, microglia also respond to proinflammatory signals generated from non-neuronal cells, including immune cells. Therefore, the crosstalk between nociceptor neurons and immune cells and between immune cells in the PNS and CNS is a primary factor affecting both acute and chronic inflammation^33–35^. Accordingly, in vitro screening of compounds in macrophage or microglial cells has been used to identify the potential analgesic compounds. Hence, in this study, curcumin and metformin were first evaluated in vitro, in lipopolysaccharide (LPS)-induced RAW 264.7 macrophage and BV-2 microglial cells to determine the nature of the interaction between compounds in peripheral and central pathways of pain.

**Figure 2:**
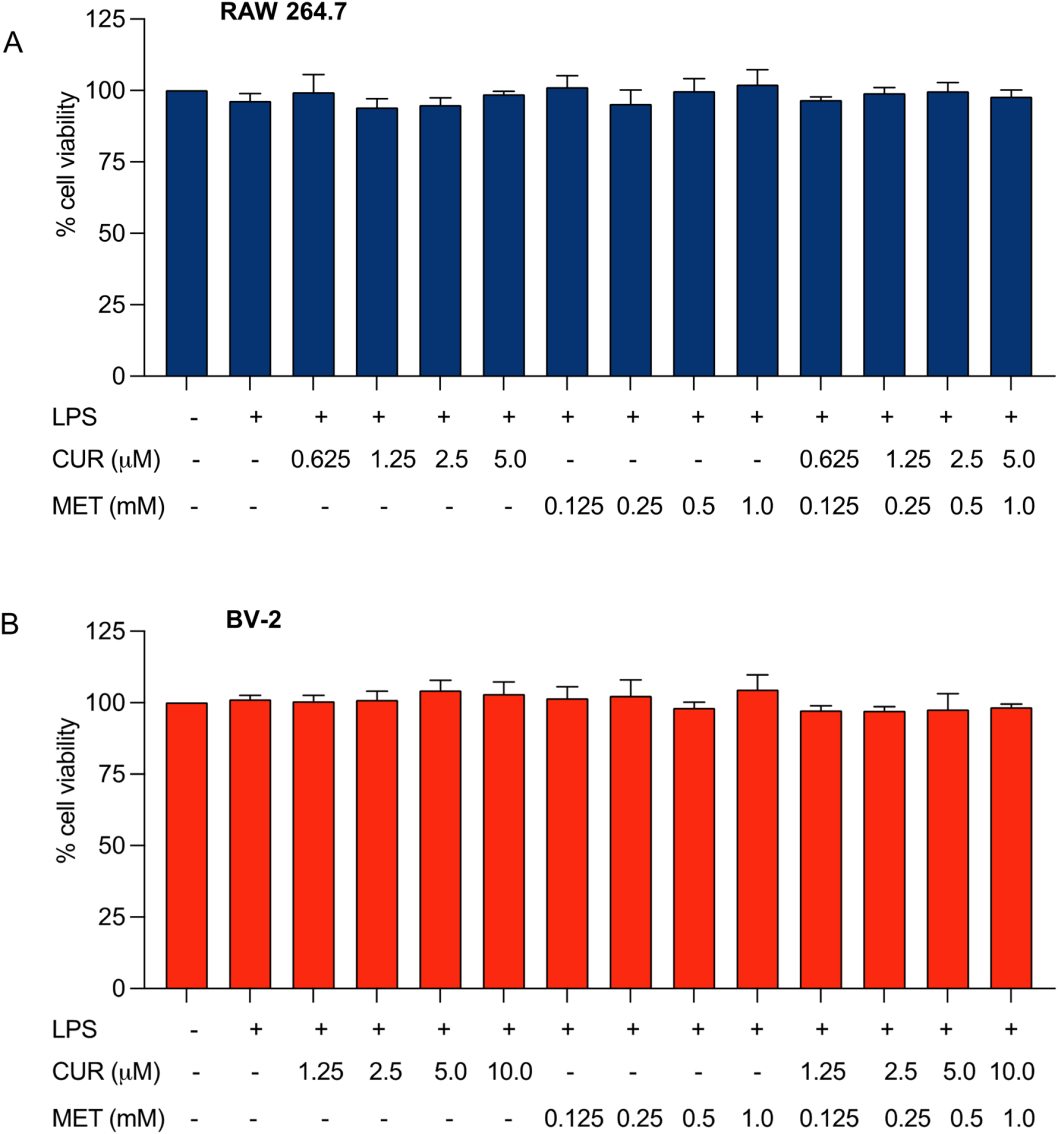
Cytotoxicity profile of LPS-stimulated RAW 264.7 macrophage (**A**) and BV-2 microglial (**B**) cells with or without treatment of curcumin (CUR), metformin (MET), or their combination. RAW 264.7 macrophages were treated with 0.625–5 µM of CUR, 0.125–1 mM of MET, or their combination at a 1:200 CUR: MET ratio for 12 h, whereas BV-2 cells were treated with 1.25–10 µM of CUR, 0.125–1 mM of MET or their combination at a 1:100 ratio of CUR: MET for 24 h. The cell viability was determined by MTT assay and expressed as a percentage of the control. Experimental data are presented as mean ± SD, n = 3 independent experiments.

Initially, the maximum non-cytotoxic concentrations of each compound alone were determined, which were 5 µM and 10 µM for curcumin in RAW 264.7 and BV-2 cell lines, respectively, and 1 mM for metformin in both cell lines. Concentrations for combination treatment were determined based on their safety concentrations: curcumin and metformin ratios of 1:200 and 1:100 for RAW 264.7 and BV-2 cells, respectively. As shown in Fig. 2, none of the treatments at selected concentrations caused significant cytotoxicity on LPS-stimulated RAW 264.7 and BV-2 microglial cells: the cell viability was greater than 94%.

Then the anti-inflammatory effect of curcumin and metformin in LPS-stimulated RAW 264.7 macrophage and BV-2 microglial cells was determined using the compounds alone and in combination. Treatment with LPS induces the release of nitric oxide, one of the main inflammatory mediators released at the inflammatory sites, into the culture medium. Secretion of nitric oxide (NO) is mediated via inducible nitric oxide synthase (iNOS), in which a high level of nitric oxide is identified as a marker of severe inflammation^36^. Therefore, the suppression of nitric oxide release is considered a plausible approach for the attenuation of the inflammatory process. The experiment was designed based on the Chou–Talalay method for synergy determination of drug combinations using the constant ratio drug model^37^. The nitrite concentration in the culture supernatant was determined as an indicator of nitric oxide^38^.

**Figure 3:**
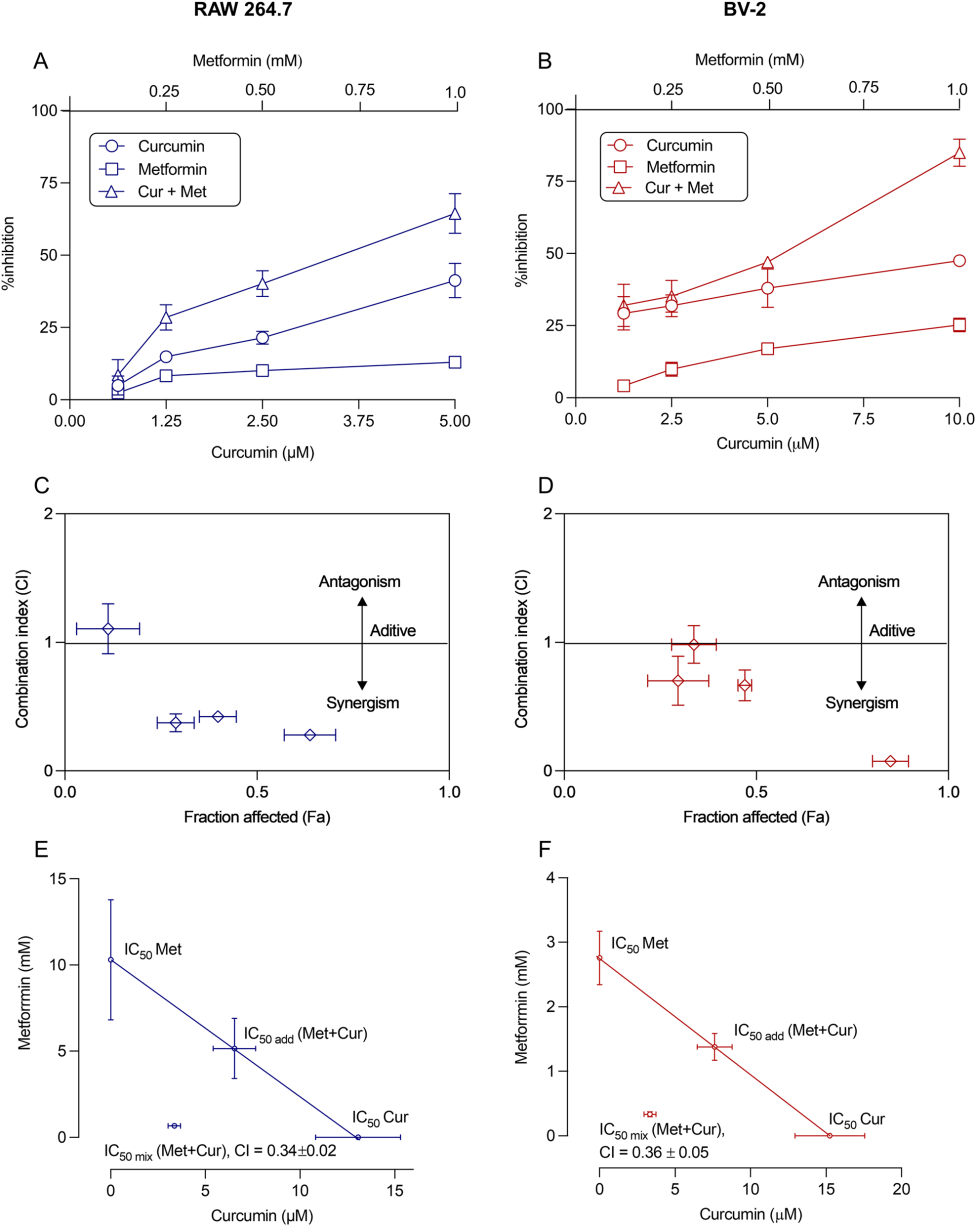
Anti-inflammatory effects of curcumin and metformin alone and in combination on peripheral and central immune cells. (**A,B**) Dose–response curves of NO inhibitory effect of curcumin and metformin alone or in combination on LPS-induced RAW 264.7 macrophage (**A**) and BV-2 microglial (**B**) cells. (**C,D**) Fraction affected (Fa)-combination index (CI) plot for the combined effect of curcumin and metformin on LPS-induced NO production in RAW 264.7 (**C**) and BV-2 (**D**) cells. Generally, CI < 1, CI = 1, and CI > 1 indicate synergistic, additive, and antagonistic interaction, respectively. (**E,F**) Normalized isobologram for the anti-inflammatory effect of curcumin and metformin on RAW 264.7 macrophage (**E**) and BV-2 microglial (**F**) cells. Experimental data are presented as mean ± SD, n = 3 independent experiments.

Curcumin, metformin, and their combination inhibited LPS-induced NO production in a concentration-dependent manner (Fig. 3A,B). When curcumin concentrations at 1.25–5 µM were combined with metformin 0.25–1 mM, a synergistic response was achieved in RAW 264.7 macrophage cells. However, in BV-2 cells, only additive or antagonistic effects were observed at lower concentrations of combination treatment except for the highest concentration of combination (10 µM CUR and 1 mM MET), which showed strong synergism (Fig. 3C,D). When the LPS-stimulated cells were treated with the highest concentration of curcumin alone (5 and 10 µM for RAW 264.7 and BV-2 cells, respectively), NO production decreased only by 41.2 ± 3.4% and 52.7 ± 3.4% in RAW 264.7 and BV-2 cells, respectively. A lower level of inhibition in NO production was observed when 1 mM of metformin alone was applied to cells: by 13.0 ± 0.8% and 26.4 ± 5.3% in RAW 264.7 and BV-2 cells, respectively. However, once the combination therapy was put in, NO release reduced remarkably: by 64.4 ± 3.9% and 89.9 ± 2.2% in RAW 264.7 and BV-2 cells, respectively.

The interaction between curcumin and metformin was then determined by calculating the combination index value according to the Chou and Talalay method, where the CI values of 1, > 1, and < 1 referred to additive, antagonistic, and synergistic interactions, respectively^37^. The CI values of the curcumin-metformin combination in RAW 264.7 and BV-2 cells were 0.28 ± 0.02 and 0.08 ± 0.01, respectively, which indicate a very strong synergism between curcumin and metformin in both the cell lines. Moreover, the IC_50_ values of the curcumin-metformin combination in both RAW cells (3.47 ± 0.35 µM CUR + 0.67 ± 0.07 mM of MET) and BV-2 cells (3.5 µM of CUR + 0.35 mM of MET) were lower than the IC_50_ values obtained for individual treatment with metformin (10.31 ± 3.48 mM for RAW.264.7 and 2.76 mM for BV-2 cells) and curcumin (13.07 ± 2.24 µM for RAW 264.7 and 8.86 µM for BV-2 cells). To further demonstrate the synergistic anti-inflammatory effect between curcumin and metformin, an isobologram was constructed. The experimentally derived IC_50_ values of curcumin and metformin in both cell lines were located below the additivity line of the isobologram, indicating a synergistic interaction between the two compounds (Fig. 3E,F).

### The combination of curcumin and metformin decreased expression levels of proinflammatory cytokines in LPS-stimulated RAW 264.7 macrophage and BV-2 microglial cells

**Figure 4:**
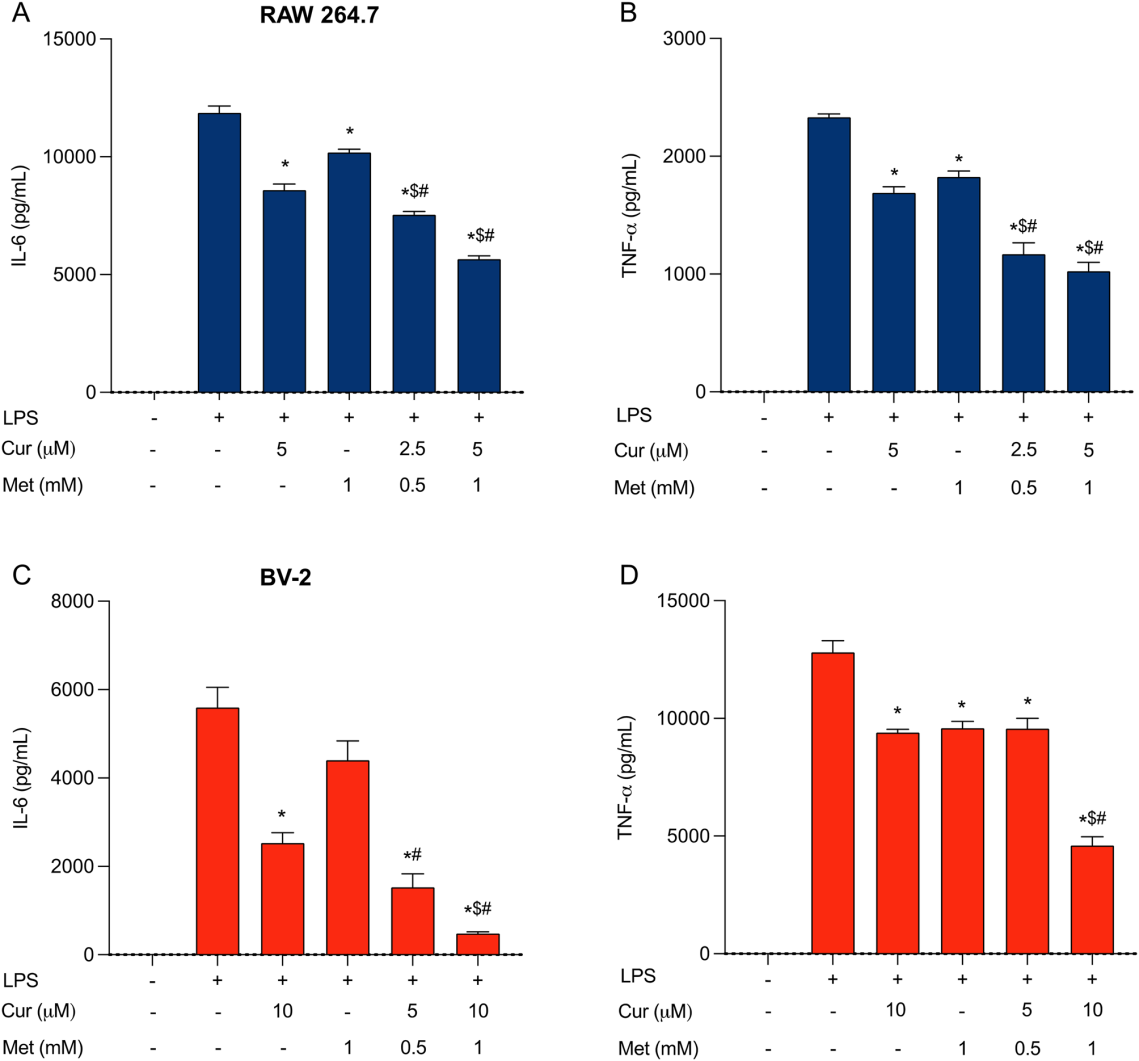
Effects of curcumin, metformin alone, and their combination on proinflammatory cytokine production in peripheral and central immune cells. (**A,B**) The effect of 5 µM curcumin, 1 mM metformin, and their combination on IL-6 (**A**) and TNF-α (**B**) expression in LPS-stimulated RAW 264.7 macrophage cells. (**C,D**) The effect of 10 µM curcumin and 1 mM metformin and their combination on IL-6 (**C**) and TNF-α (**D**) expression in LPS-stimulated BV-2 microglial cells. Data are expressed as means ± SD calculated from three independent experiments. *p < 0.05 treatments compared to LPS only, ^$^*p* < 0.05 compared to the curcumin alone and ^#^*p* < 0.05 compared to the metformin alone.

The overexpression of proinflammatory cytokines is one of the major factors governing peripheral and central pain sensitization. Hence, reducing the production of proinflammatory cytokines by both peripheral immune cells (macrophages) and central immune cells (microglia and astrocytes) is an effective approach for improving pain conditions^36,39^. Thus, we examined the effect of curcumin and metformin in combination on the release of LPS-induced proinflammatory cytokines: IL-6 and TNF-α. When RAW 264.7 macrophage cells were treated with the highest nontoxic concentrations of curcumin (5 µM) and metformin (1 mM) alone, curcumin produced 26.2 ± 1.5% inhibition of IL-6 and 25.7 ± 1.1% inhibition of TNF-α, while metformin alone produced 11.2 ± 3.1% inhibition of IL-6 and 20.4 ± 0.2% inhibition of TNF-α. However, treatment with curcumin and metformin in combination (5 µM and 1 mM, respectively) exerted significantly higher inhibition of cytokine production compared to the individual treatment: 49.7 ± 0.5% inhibition of IL-6 and 52.4 ± 1.1% inhibition of TNF-α (Fig. 4A,B). In addition, the combination of curcumin and metformin at lower concentrations (2.5 µM and 0.5 mM, respectively) inhibited IL-6 expression in 34.6 ± 1.0% and TNF-α expression in 46.5 ± 3.8%, which are comparable with the additive inhibition of 5 µM curcumin and 1 mM metformin (Supplementary Table 1).

A similar pattern of inhibition was observed in LPS-stimulated BV-2 microglial cells. The treatment of curcumin (10 µM) and metformin (1 mM) alone leads to a moderate decrease in cytokine expression: curcumin caused 53.1 ± 3.0% and 30.4 ± 6.7% inhibition of IL-6 and TNF-α, respectively, while metformin caused 21.0 ± 3.4% and 29.3 ± 1.7% inhibition of IL-6 and TNF-α, respectively. However, the combination treatment of curcumin and metformin (10 µM and 1 mM, respectively) produced an 87.8 ± 3.8% and 64.4 ± 9.2% inhibition in IL-6 and TNF-α expression, respectively, which were significantly higher than the inhibitory effect obtained with individual treatments (Fig. 4C,D). The treatment of cells with a half lower concentration of curcumin and metformin (5 µM Cur + 0.5 mM Met) exerted 70.8 ± 10.8% inhibition of IL-6 expression and 29.5 ± 6.6% inhibition of TNF-α expression (Supplementary Table 2).

### Curcumin, metformin, and their combination alleviate formalin-induced inflammatory pain-like behaviors in mice

Following the substantial findings in the in vitro models, we further expanded the experiments to pre-clinical models using a mouse model of formalin-induced inflammatory pain. This model is commonly used to represent pain-like behaviors induced by noxious stimuli. Intraplantar administration of formalin causes a characteristic biphasic response. Phase I response is an acute pain behavior mainly caused by the immediate and extensive excitation of afferent c-fibers, and the latter phase II response results from inflammatory processes in the peripheral tissues that lead to peripheral sensitization^40^. Despite the short-term responses, phase II is also characterized by a sustained inflammatory mediator release due to the activation of spinal microglia, which leads to sensitization of projection neurons, termed central sensitization^41–43^.

**Figure 5:**
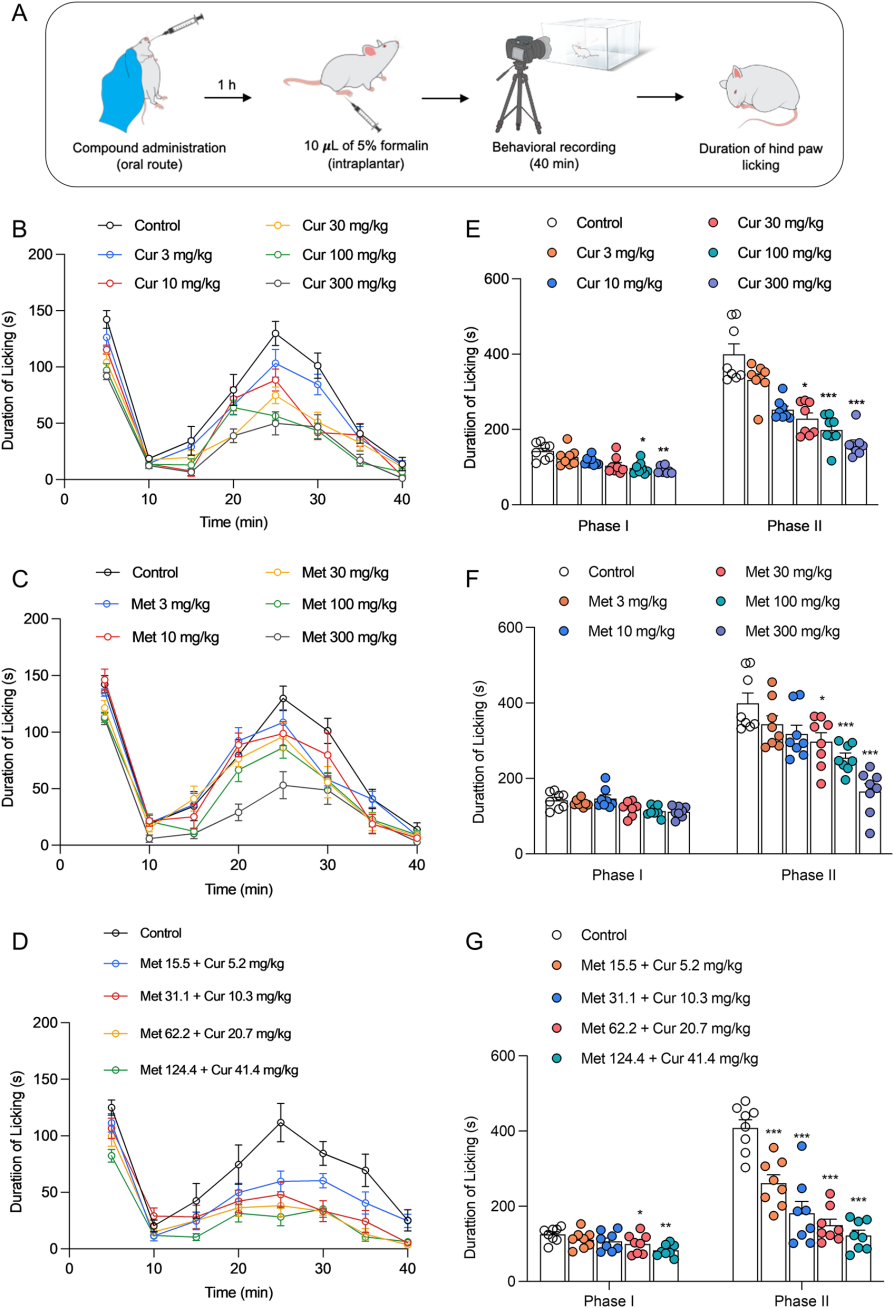
Orally administered curcumin, metformin, and their combination alleviate the formalin-induced inflammatory pain in mice. (**A**) Schematic presentation of the experimental design. (**B–D**) Time course of formalin-induced hind paw licking behavior in mice with oral curcumin (**B**), metformin (**C**), and their combination (**D**). **(E–G**) The total duration of hind paw licking during phases I and II of the pain-like behavioral response with the treatment of curcumin **(E)**, metformin **(F)**, and their combination **(G)**. Note that curcumin, metformin, and their combination dose-dependently inhibited phase II response in mice. *p < 0.05, **p < 0.01 and ***p < 0.001 compared to the vehicle-treated group. One-way ANOVA followed by Bonferroni’s post hoc test, n = 8 mice per treatment group.

Mice were pre-treated orally with different doses of curcumin or metformin (3–300 mg/kg) alone or combined at a fixed-dose ratio (1:1) of their respective ED_50_ doses. One-hour post-treatment, 10 µL of 5% formalin solution was administered subcutaneously to the plantar surface of the left hind paw, followed by behavioral observation for 40 min (Fig. 5A). The formalin-induced pain-like behavior is indicated as hind paw licking duration (Fig. 5A) and frequency (Supplementary Fig. 1). The administration of formalin produced specific pain-like behavior characterized by licking of the injected paw, observed in a distinguish biphasic response: early phase I (0–5 min) followed by an idle period (5–10 min) and thereafter, a late phase from 10 to 40 min (Fig. 5B–D). The hind paw licking behavior peaked at 0–5 min and 20–30 min post-formalin injection, which then gradually declined in all groups. As shown in Fig. 5A,B, in the early phase, both curcumin and metformin monotherapy did not show strong antinociception where they only produced significant antinociception at higher doses (100 and 300 mg/kg). However, curcumin and metformin in monotherapy and combination therapy dose-dependently inhibited the formalin-induced hind paw licking in late phase II (Fig. 5E–G). Hence, the type of interaction between two compounds was then determined by considering their effect on phase II pain-like behaviors.

The formalin-induced first and second-phase responses had unique characteristics^44^. The first phase is due to the direct activation of nociceptors by formalin (somatic pain). The second phase is due to inflammatory processes (inflammatory pain), leading to peripheral and central sensitization. Medicines that act centrally, such as narcotics, inhibit both stages equally, but those that act peripherally, such as NSAIDs and steroids, only inhibit the second phase. Thus, this model enables researchers to distinguish between somatic and inflammatory pain and central and peripheral analgesic mechanisms^45^. In this study, curcumin and metformin showed effects in both phases, wherein in phase 1, significant antinociception was only observed at higher doses (100 and 300 mg/kg). However, more potent inhibition was observed in phase II licking behaviors. This indicates the potential contribution of both the PNS and CNS to the curcumin and metformin antinociception.

### Curcumin and metformin produce synergistic antinociception in formalin-induced mice

**Figure 6:**
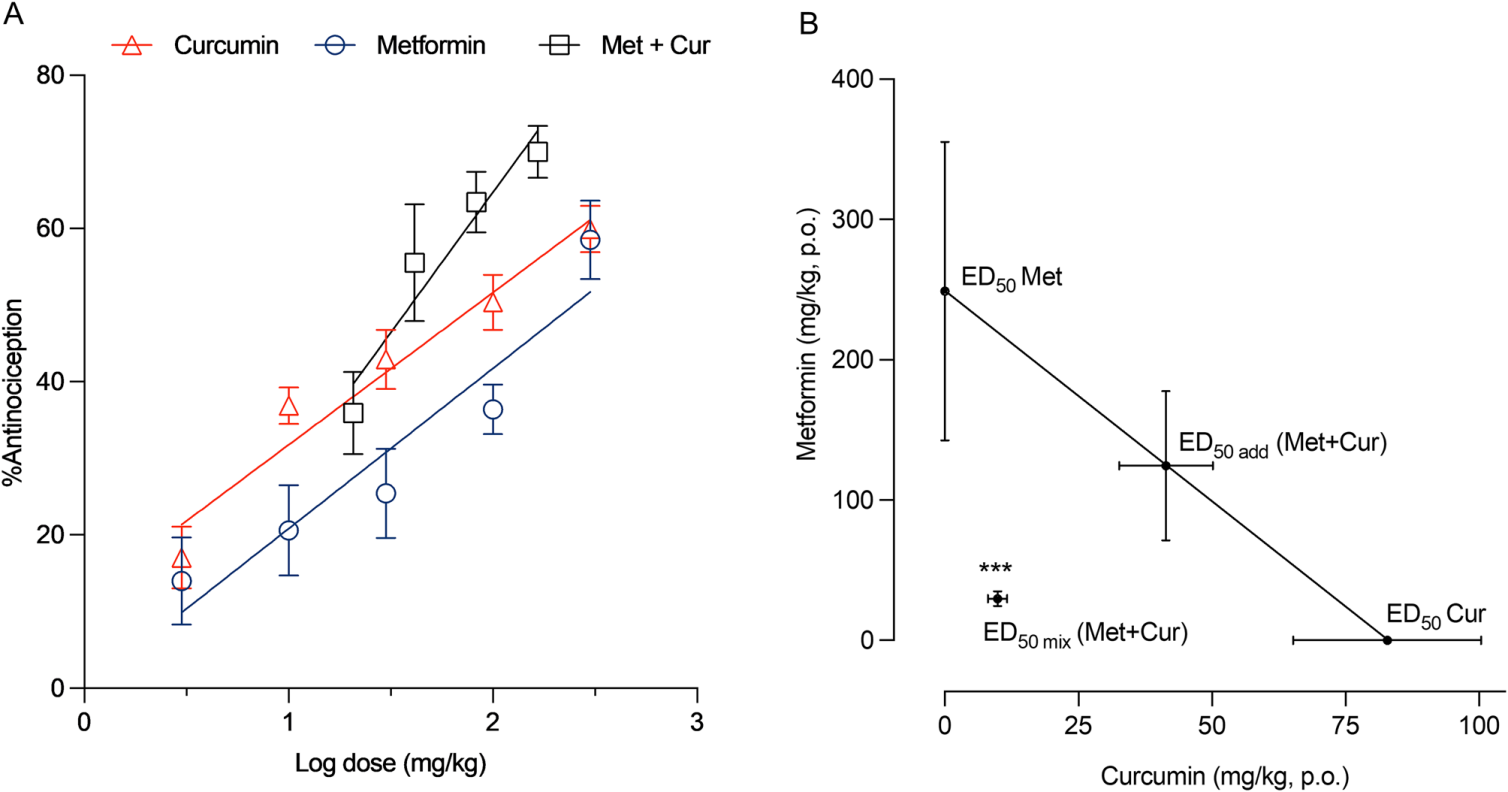
Isobolographic analysis of the antinociceptive interaction between curcumin and metformin in phase II of the formalin test. (**A**) Linear regression of log dose–response curves of curcumin, metformin, and curcumin-metformin combination. (**B**) The isobologram of the curcumin-metformin interaction at the fixed-dose ratio of 1:1. The straight line connecting the ED_50_s of curcumin and metformin represents the additive line. The horizontal and vertical bars represent the SEM. ED_50_ (Cur) and ED_50_ (Met) correspond to the ED_50_s obtained with individual treatment of curcumin and metformin, respectively. ED_50 add_ and ED_50 mix_ correspond to the theoretical and experimentally derived ED_50_s of curcumin and metformin combination. The ED_50 mix_ value lies far below the additive line, suggesting significant synergism. ***p < 0.001 compared to the ED_50 add,_ by student’s *t*-test.

Interaction between curcumin and metformin with respect to the antinociceptive effect exerted by the treatments in the late phase of the formalin test was analyzed according to the method described by Tallarida et al*.*^46^*.* Initially, the log dose–response curves for the effect of acute oral administration of curcumin, metformin, and curcumin-metformin combination were constructed (Fig. 6A). Curcumin and metformin showed dose-dependent antinociceptive effects where the maximum dose evaluated (300 mg/kg) demonstrated the maximal antinociception: 60% for curcumin and 58% for metformin. The doses exerting 50% antinociception (ED_50_) were established at 82.8 ± 17.6 mg/kg and 248.9 ± 106.5 mg/kg for curcumin and metformin, respectively assuming a linear-logarithmic model of the dose–response curves. As shown in Fig. 6A, the dose–response curve obtained for the curcumin and metformin co-treatment at a 1:1 fixed ratio showed a dose-dependent effect, and the curve shifted to the left. There was a remarkable reduction in the ED_50_ to 39.6 ± 7.1 mg/kg (ED_50 mix_) and 70% maximum antinociception. The slopes of regression lines for curcumin and metformin monotherapy were 19.9 ± 2.2 and 20.9 ± 3.4, respectively. The statistical analysis indicated the parallelism of the dose–response curves (*p* > 0.05, student’s *t*-test). Hence, the type of interaction between compounds was determined using standard type I isobololographic analysis.

The isobologram of curcumin-metformin combination indicated synergistic interaction between curcumin and metformin (Fig. 6B) as the experimentally derived ED_50 mix_ located below the theoretical additive line connecting ED_50_ values of curcumin and metformin monotherapy. Table 1 presents the theoretical additive and experimentally derived ED_50_ values for the combination. As indicated in the table, the experimentally derived ED_50 mix_ was substantially reduced by 76% than the theoretically presumed ED_50 add_ (*p* < 0.001, student’s *t*-test). In addition, the combination index of the curcumin-metformin combination was 0.24. Altogether, these results suggest the synergistic interaction between curcumin and metformin in inhibiting phase II pain-like behaviors of formalin-induced mice at the 50% effect level.

**Table 1:** 50% antinociceptive doses and interaction index of curcumin, metformin, and curcumin–metformin combination in phase II of mouse formalin test.

Monotherapy		ED_50_ ± SEM (mg/kg)		
Curcumin		82.8 ± 17.6		
Metformin		248.9 ± 106.5		

Combination therapy (1:1)	Ratio	ED_50 add_ ± SEM (mg/kg)	ED_50 mix_ ± SEM (mg/kg)	CI
CUR + MET	1:3	165.8 ± 62.0	39.6 ± 7.1***	0.24
Curcumin		41.4 ± 8.8	9.9 ± 1.8	
Metformin		124.4 ± 53.2	29.7 ± 5.3	

The ED_50_ values were determined from linear regression analysis of the log dose–response curves. The theoretical ED_50_ (ED_50 add_) was calculated based on the dose–response curves of the curcumin and metformin monotherapy. The experimental ED_50_ (ED_50 mix_) was determined by the experimentally derived dose–response curve of the curcumin–metformin combination treatment. The ED_50_s are presented with their respective S.E.M. values.

*CI* combination index.

***p < 0.001 compared to the ED_50 add_ (student’s *t-*test).

Previous studies reported curcumin synergism with other analgesic drugs, including diclofenac and pregabalin, in animal models of nociceptive pain^47,48^. These previous findings, along with our study findings, indicate the potential of curcumin to exert synergistic multimodal analgesia with other drugs, which could be due to the diverse and complementary mechanisms of action of curcumin. The analgesic effect of curcumin was explained by its ability to attenuate neurotransmitters related to pain (substance P), suppress the immune response, and prostaglandin E2 production by suppressing cyclooxygenase-2 (COX-2), modulating purinergic and chemokine receptors, and activate the opioid system^49^. The pharmacodynamic interaction between curcumin and metformin is plausible as metformin shows a distinct mechanism of analgesia to curcumin: activation of AMPK, opioidergic mechanisms, and suppressing peripheral and central inflammation^6,13,14,50^. Consequently, metformin showed synergistic analgesia with several other analgesics, including ibuprofen, aspirin, tramadol, and pregabalin^51^.

Administration of formalin into rodents’ hind paws is also associated with excitation of sensory neurons via direct activation of cation channels TRPA1/TRPV1^52,53^. Moreover, effective attenuation of TRP channels by both metformin and curcumin has been reported. Curcumin was found to regulate TRPV1 channels in vivo via antagonizing their activation and inhibiting phosphorylation^25,54^. In addition, metformin was also found to modulate TRPV1 channels in a murine model of bone cancer via decreased ASIC3 and TRPV1 expression^55^. Therefore, inhibition of TRP receptors may also have a major contribution to the curcumin-metformin synergistic antinociception, which needs to be confirmed in future studies.

Moreover, the synergistic analgesia obtained between curcumin and metformin could also be due to the pharmacokinetic interaction. Oral administration of curcumin was reported to inhibit several drug-metabolizing enzymes, including cytochrome P450: CYP3A4, glutathione-S-transferase, and UDP-glucuronosyltransferase. Hence, oral curcumin acts as a bioenhancer for the drugs metabolize via cytochrome P450 enzymes, including morphine, acetaminophen, and digoxin^56^. Metformin, on the other hand, has a limited probability of eliciting pharmacokinetic interactions since it isn’t metabolized or bound to plasma proteins in substantial amounts^57^. Hence, in this study, pharmacokinetic interactions were not evaluated yet cannot be excluded.

### The underlying mechanism of curcumin-metformin combination by network pharmacology analysis

**Figure 7:**
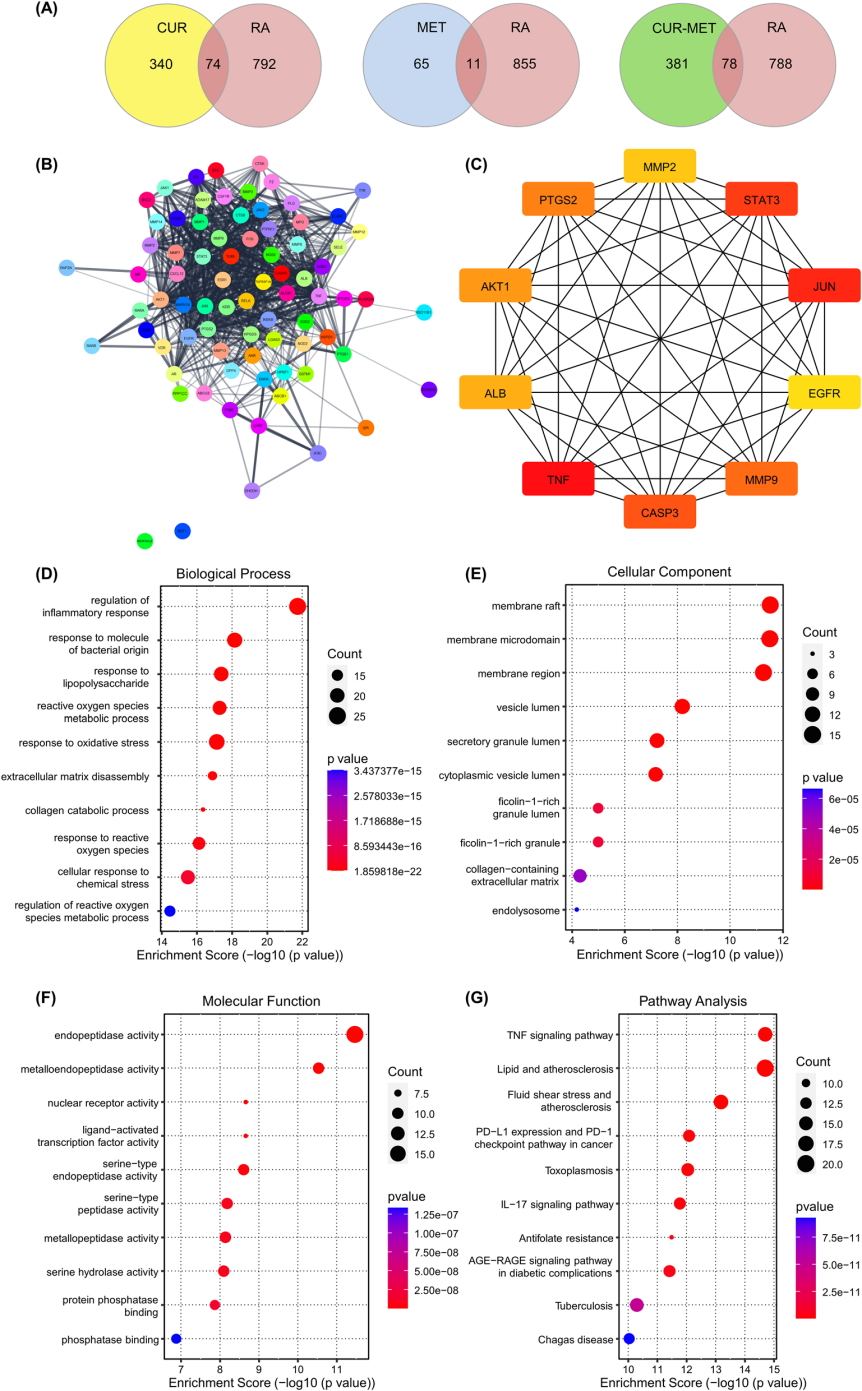
Network pharmacology analysis of underlying mechanisms of curcumin-metformin combination in inflammatory pain. (**A**) The intersection between the test compounds (curcumin, metformin, and curcumin-metformin combination) and rheumatoid arthritis-related targets. (**B**) Protein–protein interaction of 76 intersection genes (**B**) and the top 10 hub gene-network interactions (**C**). Gene ontology (**D–F**) and KEGG (**G**) enrichment pathway analyses. *CUR* curcumin, *MET* metformin, *MET-CUR* metformin-curcumin combination, *GO* Gene Ontology, *KEGG* Kyoto Encyclopedia of Genes and Genomes.

In the present study, the potential underlying mechanism of the curcumin-metformin combination was investigated using network pharmacology. Network pharmacology integrates pharmacology and network biology. It is an approach to providing a comprehensive overview of the interactions between compounds, targets, and diseases in a holistic way^58^. As shown in Fig. 7, there are 414, 76, and 459 potential targets for curcumin, metformin, and curcumin-metformin combination, respectively. In addition, 866 targets of rheumatoid arthritis (RA) associated genes were retrieved from the databases. The Venn diagram shows that the intersection target genes between rheumatoid arthritis and curcumin, metformin, and the curcumin-metformin combination were 74, 11, and 78, respectively (Fig. 7A). The interactions between compounds and inflammatory pain-related genes were also constructed using the Cytoscape. As shown in network interaction, some genes were targeted by both curcumin and metformin (Supplementary Fig. 2).

Intersection targets between curcumin-metformin combination and inflammatory pain-related diseases (RA) were analyzed for protein–protein interactions using the STRING database. The results demonstrate complex interactions between the involved genes. In the network, there were 78 nodes and 785 edges constructed. Furthermore, the PPI network was analyzed using CytoHubba (maximal clique centrality (MCC) analysis to obtain 10 top interactions between genes. The top 10 genes include AKT1, ALB, CASP3, EGFR, JUN, MMP2, MMP9, PTGS2, STAT3, and TNF (Fig. 7C). These targets potentially play a major role in the effects of the curcumin-metformin combination on inflammatory pain. The illustrations of biological mechanisms and pathways were obtained after GO and KEGG enrichment analyses (Fig. 7D–G). The top five biological processes were significantly enriched in the regulation of inflammatory processes, responses to bacterial molecules, lipopolysaccharide, oxygen species metabolic process, and oxidative stress. These biological processes play a major role in inflammatory pain, indicating the potential ability of the curcumin-metformin combination to alleviate inflammatory pain (Fig. 7D). Furthermore, the top five cellular components were significantly enriched in the membrane raft, membrane microdomain, membrane region, vesicle lumen, and cytoplasmic vesicle lumen (Fig. 7E).

For the molecular function, the activities of endopeptidase, metalloendopeptidase, nuclear receptor, ligand-activated transcription factor, and serine-type peptidase were substantially enriched (Fig. 7F). To illustrate the potential pathways of curcumin-metformin in the treatment of inflammatory pain, the potential targets were analyzed for KEGG pathway enrichment analysis. The top five pathways include TNF signaling pathway, lipid and atherosclerosis, fluid shear stress and atherosclerosis, PD-L1 expression and PD-1 checkpoint pathway in cancer, and toxoplasmosis (Fig. 7G). Here, we found that inflammatory pathways, especially the TNF-signaling pathway, are the main pathways involved in the anti-inflammatory effects of the curcumin-metformin combination. The TNF-signaling pathway has been associated with inflammatory pain, especially in rheumatoid arthritis. The blockage of TNF-alpha resulted in the improvement of pain-like behaviors^59^. Therefore, the ability of the combination to suppress inflammatory pain could be attributed to its ability to modulate immune cells and inflammatory pathways.

### Synergistic attenuation of pain-like behaviors by curcumin and metformin is not accompanied by CNS side effects

**Figure 8:**
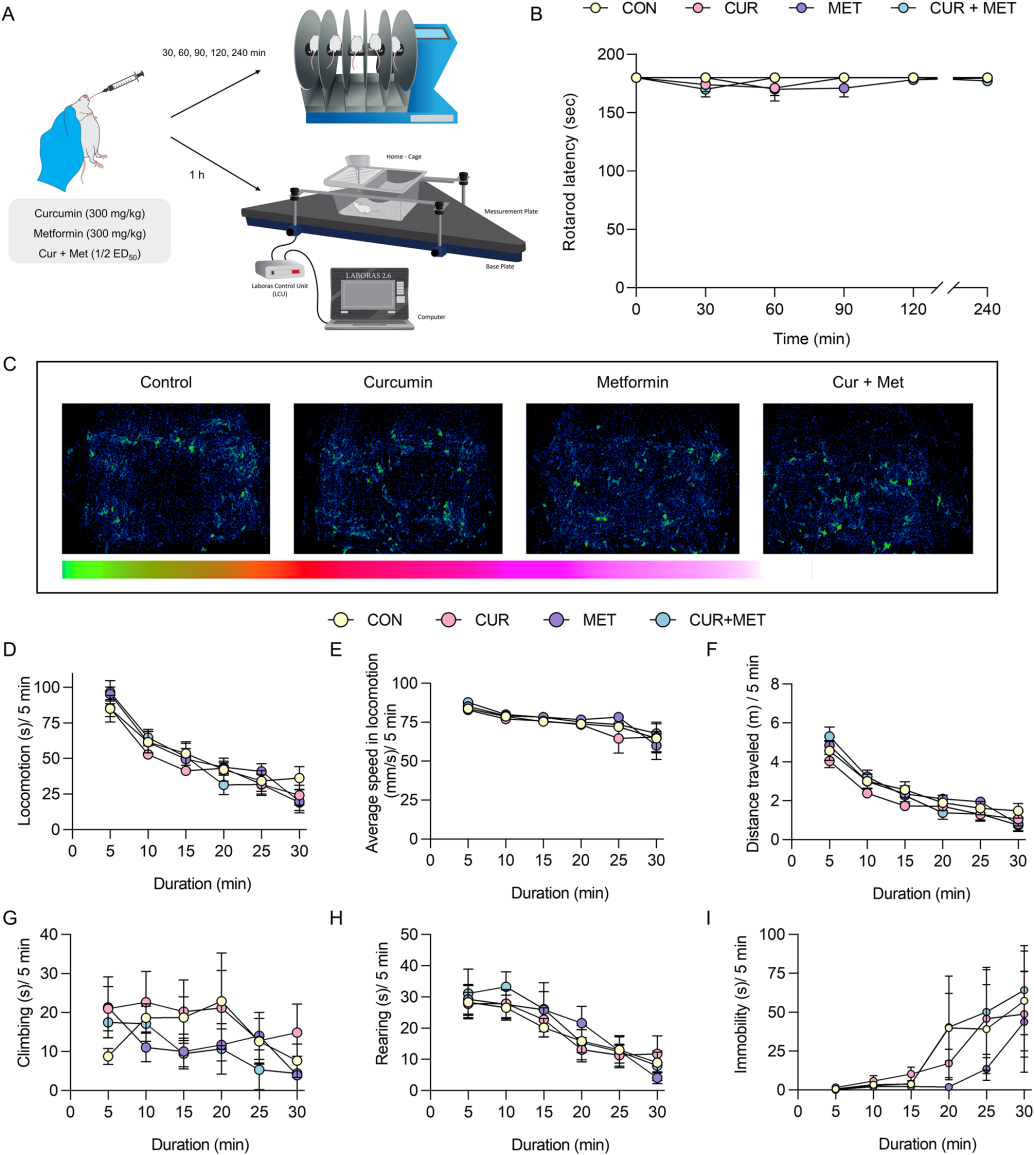
Effects of curcumin and metformin alone or in combination on forced and spontaneous locomotor activity of naïve mice. (**A**) Schematic presentation of the experimental design. (**B**) Effect of treatments on rotarod performance (n = 6/group). (**C–I**) Short-term locomotive behaviors, measured by LABORAS 1 h post-treatment for 30 min (n = 8 mice/group). (**C**) Position distribution of mouse inside the home cage. (**D**) Time spent on locomotion. (**E**) Average speed in locomotion. (**F**) Distance traveled in each 5 min. (**G–I**) Time spent on climbing, rearing, and immobility, respectively. Data are presented as mean ± S.E.M. Short-term locomotive behavioral data were analyzed over 5-min intervals during 30 min test session.

In light of the antinociceptive synergism between curcumin and metformin in the formalin test, we evaluated the effects of individual compounds and their combination on motor coordination and short-term locomotor activity in naïve male-ICR mice. Measurement of locomotor activity allows us to determine whether the combination has potential side effects, either sedative or CNS stimulative^60,61^. The compounds in monotherapy or combination therapy were tested at the highest doses used in the formalin test (Fig. 8A). The effect of drug combination on forced locomotive behavior was evaluated using the rotarod test. The mouse’s ability to remain on a rotating rod (18 rpm) was measured periodically at 30-, 60-, 90-, 120- and 240-min post-treatments. As shown in Fig. 8B, curcumin and metformin alone or in combination showed no effect on motor coordination, balance, and muscle relaxation in the animals. Moreover, the effect of compounds on short-term spontaneous locomotor activity was measured by using an automated home-cage monitoring system, Laboratory Animal Behavior Observation, Registration and Analysis System (LABORAS). Compounds alone or combined were orally administered 1 h before the behavioral measurements, and the behavioral measures were taken for 30 min (Fig. 8A). Mice treated with either curcumin and metformin alone or in combination explored throughout the home cage as indicated by position distribution in Fig. 8C. Neither curcumin and metformin monotherapy nor curcumin-metformin combination caused significant alterations in short-term spontaneous locomotive behaviors (locomotion, climbing, and rearing) compared to that of the control group (*p* > 0.05) (Fig. 8D–I). The vehicle-treated mice engaged in locomotor behavior mainly during the first 15 min of the experiment. In contrast, during the latter period of the experiment (15–30 min), time spent in immobility was increased. This behavioral pattern is most likely due to the exploratory behaviors followed by adaptation to the new home cage environment. The same behavioral stereotype was observed with the treatment of curcumin and metformin alone or in combination, with no significant difference in behaviors compared to the vehicle-treated group.

### Curcumin, metformin, and their combination showed no significant effect on the general behavior and well-being of mice

**Figure 9:**
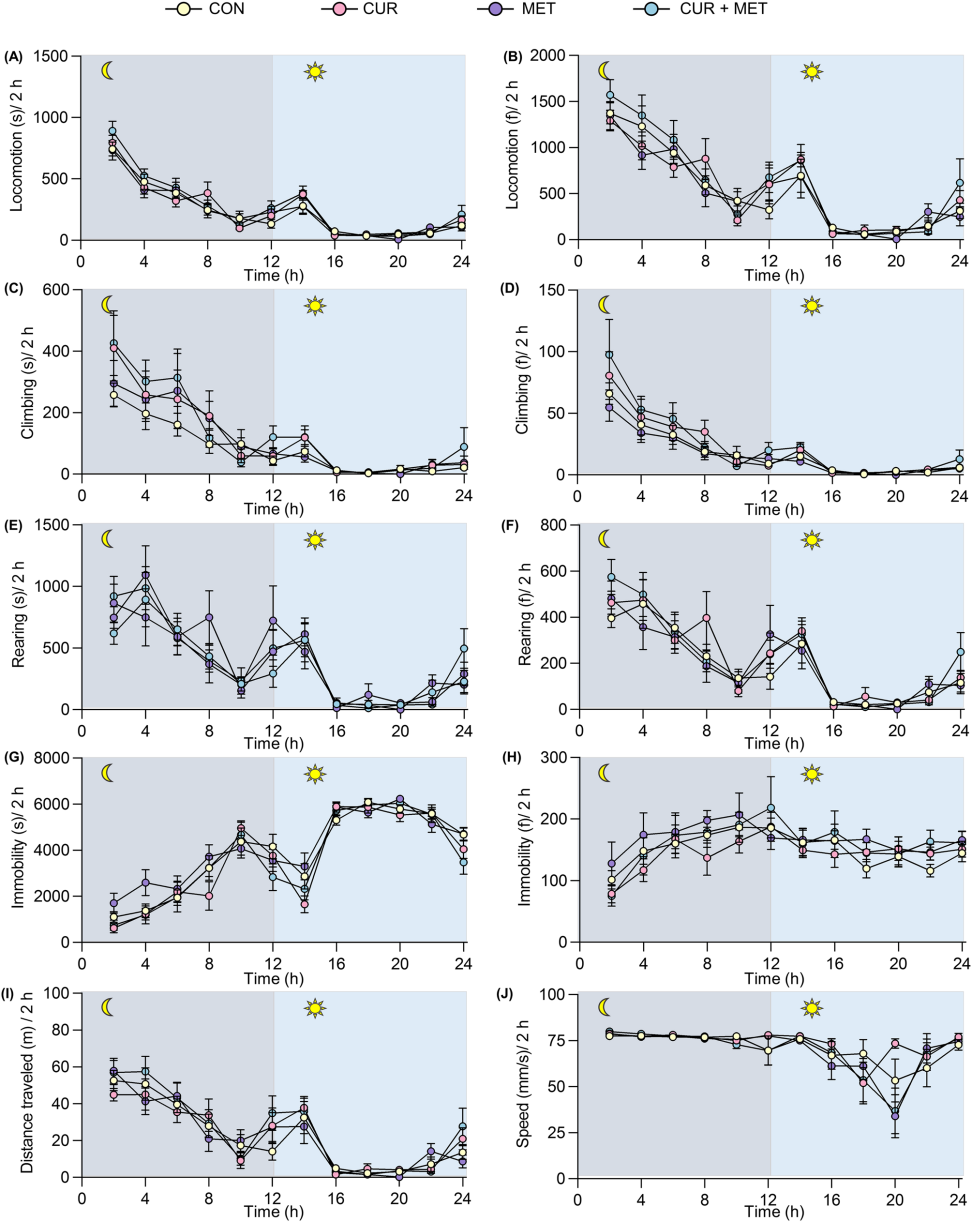
Effects of curcumin and metformin alone or in combination on the general behavior of naïve mice. Behavioral measures were recorded for 24 h post-treatment using LABORAS. (**A,B**) Time spent on locomotion (**A**) and frequency of its occurrence (**B**). (**C,D**) Time spent on climbing (**C**) and frequency of its occurrence (**D**). (**E,F**) Time spend on rearing behavior (**E**) and frequency of its occurrence (**F**). (**G,H**) Time spent on immobility (**G**) and frequency of its occurrence (**H**). (**I**) Distance traveled by mouse in the cage. (**J**) Average speed in locomotion. Data are presented as mean ± S.E.M (n = 10 mice/group). Behavioral data were analyzed over 2 h intervals during 24 h test sessions.

The general behavior and well-being data in rodents can be translated to CNS side effects in humans^62^. The different behaviors of rodents, including locomotor activity and rearing, immobility, and food intake/body weight, are used to define dizziness, somnolence, and nausea in humans, respectively^62^. Hence, curcumin and metformin alone and their combination were evaluated on LABORAS for 24 h to assess their effects on the general behavior of mice. Mice were administered with either curcumin and metformin alone or in combination at the highest doses-tested, placed individually in the LABORAS home cages, and behavioral measures were recorded for 24 h. Mice treated with the vehicle spent more time on mobile behaviors during the nighttime compared to the daytime (Fig. 9). This behavioral pattern is expected as the mice are nocturnal animals. The time spent on and frequency of mobile behaviors, including locomotion, climbing, and rearing, gradually declined for 12 h. (18.00–6.00), slightly increased at the beginning of the daytime (6.00–8.00), and nearly no mobility was observed thereafter, which was then started to increase by the end of the test period (16.00–18.00). In line with that, immobility was lower during the nighttime and higher during the daytime (Fig. 9A–H). Distance traveled by vehicle-treated mice inside the cage also showed the same pattern with mobile behaviors (Fig. 9I). Moreover, the vehicle-treated mice showed a maximum average speed of 77 mm/s, which remained static during the nighttime, gradually declined during the daytime, and then reached the same constant level by the end of the experiment. The same behavioral pattern was observed with the treatment of compounds in monotherapy or combination (Fig. 9). Neither individual nor combination treatment significantly affected on long-term spontaneous locomotive behavior in mice observed during the day and nighttime.

**Figure 10:**
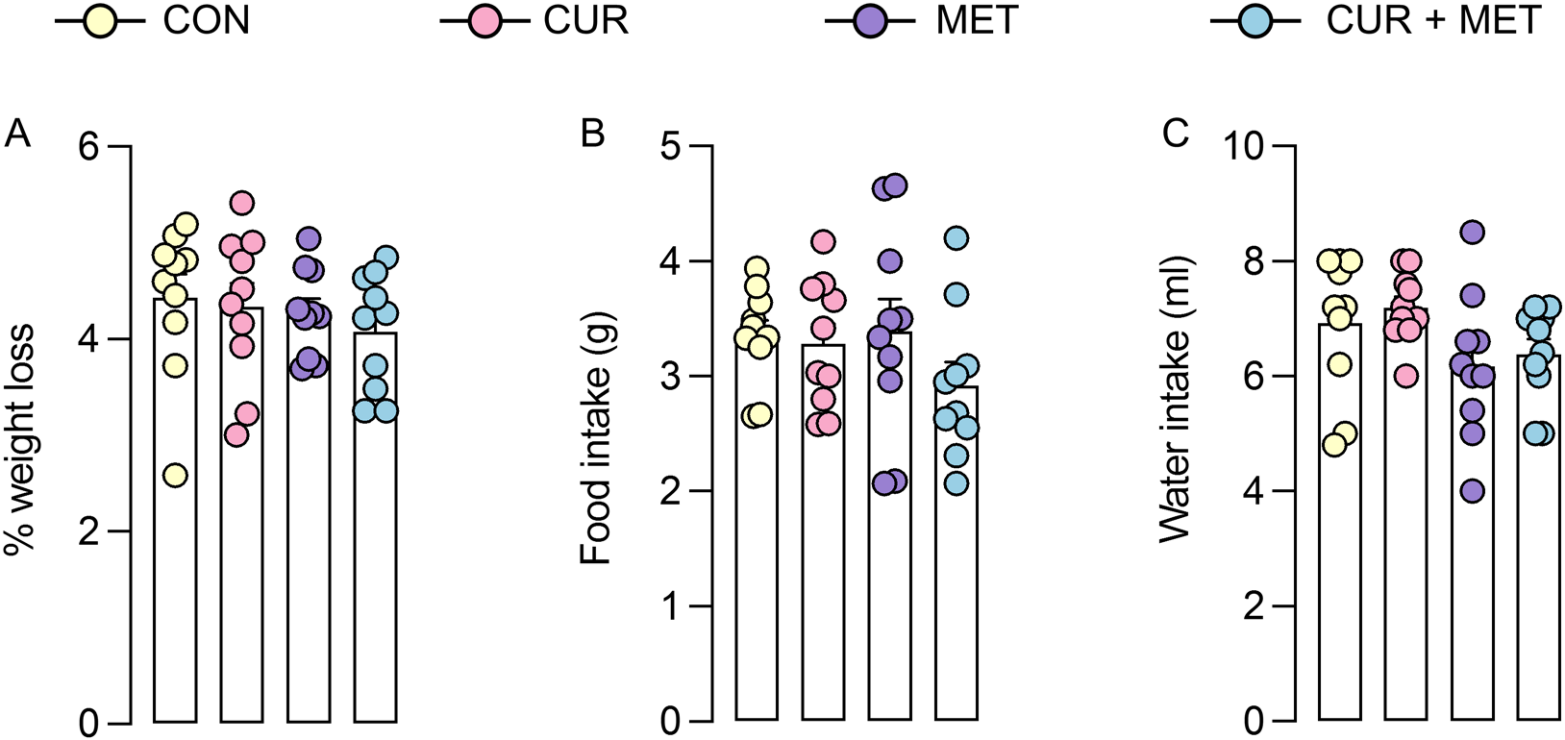
Percentage weight loss, food intake, and water intake of mice after 24 h treatment with curcumin and metformin alone or in combination. The weight of the mice and food and water volume supplied to each cage were measured pre-, and post-test (24 h). The percentage weight loss (**A**) and food intake (**B**), and water intake (**C**) were calculated and presented as mean ± S.E.M (n = 10 mice/group).

The body weight and food and water intake are also considered measures of general well-being in rodents. Hence, we evaluated the effects of compounds in monotherapy or combination on those parameters during the 24 h LABORAS test period. As indicated in Fig. 10A, mice in the vehicle group showed an average weight loss of 4.4%, wherein treatment groups showed no significant difference in the weight loss compared to the vehicle-treated group. The weight loss observed in all the treatment groups, including the vehicle-treated group, could be attributed to the isolation of animals during the LABORAS experiment. Generally, mice are identified as social creatures. During this experiment, mice must be individually placed in the LABORAS cage where they were originally housed as 5 mice/cage. Hence, the isolation of mice from their peers could stress out the animals leading to weight loss. Moreover, the vehicle-treated group showed average food and water intake of 3.6 g and 6.9 mL, respectively, for 24 h. The values obtained with the treatment of curcumin and metformin alone or in combination showed no significant difference from the vehicle-treated group (Fig. 10B,C). These results indicate that curcumin and metformin in monotherapy or combination had no effect on the general well-being of mice.

Most of the available analgesics are reported to possess CNS side effects. For example, NSAIDs are associated with drowsiness, cognitive dysfunction, and psychotic disorders^63,64^; opioids are associated with psychomotor and cognitive impairment, drowsiness, and sleep disturbances^65,66^. In addition, gabapentanoids are reported to cause sedation and cognitive effects^67^, and CNS side effects of cannabinoids are depression, sedation, euphoria, and psychosis^68^. Thus, analgesics with a higher safety margin are a constant challenge in drug development. Interestingly, our study reports no potential CNS side effects of curcumin and metformin in combination, suggesting its possible use in clinical trials. However, further studies in humans are warranted to ensure the efficacy and safety of the curcumin-metformin combination in the management of pain.

## Conclusion

In summary, this study suggests for the first time that curcumin combined with metformin exerts synergistic anti-inflammatory effects in both in vitro and in vivo conditions. Curcumin synergistically augmented the inhibition of nitric oxide and proinflammatory cytokines by metformin both in RAW 264.7 macrophage and BV-2 microglial cells. Besides, the in vivo experiments through formalin-induced mice verified the synergistic analgesic effects. Moreover, the combined therapy using curcumin and metformin showed no considerable CNS adverse effects in naïve mice. Hence, this study supports the possibility of combined use of curcumin and metformin in the treatment of pain with the least amount of medication while taking the easiness of administration, cost of the therapy, and side effect profile of medicines into the account.

## Methods

### Cell culture

RAW 264.7 macrophage cells (ATCC, Rockville, MD, USA) and BV-2 microglial cells (Accegen Biotechnology, New Jersey, USA) were cultured in 75 cm^2^ flasks in Dulbecco’s modified Eagle’s medium (DMEM) supplemented with 10% fetal bovine serum (FBS), 1% penicillin–streptomycin (Sigma-Aldrich, St. Louis, MO, USA). Cultured flasks were incubated at 37 °C in a 5% CO_2_ humidified atmosphere. The medium was replaced every 2–3 days, and cells were sub-cultured by trypsin treatment twice a week. RAW 264.7 macrophage (200,000 cells/well) and BV-2 microglial cells (150,000 cells/well) were seeded in 24-well plates in DMEM supplemented with 10% FBS and incubated overnight. On the next day, cells were pre-treated for 2 h with curcumin, metformin, or combination, followed by incubation with lipopolysaccharide (LPS, Sigma-Aldrich, St. Louis, MO, USA). RAW cells were incubated with 100 ng/mL LPS for 12 h, and BV-2 cells with 1 µg/mL LPS for 22 h. Then the culture supernatant was collected to analyze nitrite level and cytokine production by NO assay and ELISA, respectively. Curcumin was kindly provided by the Natural Products for Ageing and Chronic Diseases Research Unit, Faculty of Pharmaceutical Sciences, Chulalongkorn University, and metformin by the Siam Bheasach Co. Ltd. (Bangkok, Thailand). All experiments were performed in triplicate, and results are mentioned as mean ± SD from three independent experiments.

### Cytotoxicity assay

Twenty-four hours after preconditioning of seeded cells, the medium was replaced with 500 µL of serum-free DMEM media containing different concentrations of curcumin (1.25–20 µM) and metformin (0.25–10 mM) or their combination. After 24 h treatments, media were replaced by MTT (3-(4, 5-dimethylthiazol-2-yl)-2, 5-diphenyltetrazolium bromide) solution (0.5 mg/mL in PBS), and the plates were incubated at dark for 2 h at 37 °C with 5% CO_2_ humidified atmosphere. Then the MTT containing media was removed, and the formed formazan crystals were dissolved by adding DMSO to each well. Finally, the plates were shaken for 10 min in the dark, followed by an absorbance measurement at 570 nm using a microplate reader.

### NO assay

The nitrite production in the culture supernatant was determined by the Griess reaction. Briefly, 100 µL of culture supernatant was transferred to a 96-well plate, and 50 µL of 1% [w/v] sulfanilamide was added and incubated for 5 min in the dark. Then, 50 µL of 2.5% [w/v] *N*-1*-*Napthylenediamine dihydrochloride was added and incubated for 5 min in the dark. The absorbance was recorded at 520 nm. The concentration of NO was calculated by comparison with a standard curve of sodium nitrite. The percentage inhibition of NO production in treated cells was expressed as the percentage of absorbance of LPS-treated cells, considered 100% NO production.

### Median-effect analysis: computerized simulation by CompuSyn

The type of interaction between curcumin and metformin on RAW 264.7 and BV-2 cells was determined according to the median-effect principle described by Chou et al.^37^ using the CompuSyn software^69^. In Chou and Talalay method, the interaction between compounds in the mixture is determined based on the median effect equation, which describes dose–effect relationships:

1 $${\text{F}}_{{\text{a}}} /{\text{ F}}_{{\text{u}}} = \, \left[ {{\text{C }}/{\text{ C}}_{{\text{m}}} } \right]^{{\text{m}}} ,$$

where F_a_ is the fraction affected by C, F_a_ ranges from 0 to 1, F_a_ = 0 and 1 represent 0% and 100% inhibition of NO production compared to the LPS control, respectively; F_u_ is the fraction unaffected (F_a_ + F_u_ = 1); C is the concentration of compound; C_m_ is the concentration required to inhibit the NO production by 50%, and m is the sigmoidicity coefficient of the dose–effect curve. The combination index (CI) that indicates the interaction between two compounds is then determined using the following formula:

2 $${\text{CI }} = \, \left[ {\text{C}} \right]_{{1}} /\left[ {{\text{C}}_{{\text{x}}} } \right]_{{1}} + \, \left[ {\text{C}} \right]_{{2}} /\left[ {{\text{C}}_{{\text{x}}} } \right]_{{2}} ,$$

where C_1_ and C_2_ are the concentrations of compound 1, and compound 2 in combination required to produce x% of effect; [C_x_]_1_ and [C_x_]_2_ are the concentrations of individual compounds required to produce the same x% effect. The type of interaction between compounds was determined by the Fa-CI plot. CI values of 1, > 1, and < 1 referred to additive, antagonistic, and synergistic interactions.

### ELISA test

The expression levels of TNF-α and IL-6 in the culture supernatant were measured using commercial enzyme-linked immunosorbent assay (ELISA) kits, as indicated by the manufacturer (BioLegend, San Diego, CA, USA). The absorbance was measured at 450 nm. The concentration of cytokines in the culture supernatant was determined from their respective standard curves.

### Animals

Male ICR mice at the age of 4–5 weeks were purchased from Nomura Siam International Co., Ltd., Bangkok, Thailand. Animals were housed (4–5 mice per cage) under controlled temperature (24 ± 2 °C), humidity (60 ± 10%) and light (12:12 h light/dark cycle), and free access to food and water at the animal facility, Faculty of Pharmaceutical Sciences, Chulalongkorn University, Thailand. Animals were allowed to acclimatize to laboratory conditions for at least one week before the test procedures. Ethical approval was obtained from the Institutional Animal Care and Use Committee, Faculty of Pharmaceutical Sciences, Chulalongkorn University, Bangkok, Thailand, before the commencement of the study (Protocol No. 2033006). All the tests were conducted in compliance with the ARRIVE guidelines (Animal Research: Reporting of In Vivo Experiments).

### Drugs and treatments

In treatment groups, curcumin or metformin was orally administered at doses of 3, 10, 30, 100, and 300 mg/kg; mice in each control group received 0.5% carboxymethylcellulose (CMC). To determine the interaction between curcumin and metformin, mice were then administered with different doses of curcumin and metformin mixed at a fixed ratio (1:1) of their respective ED_50_ dose for the individual treatment: 1/16, 1/8, 1/4, and 1/2 (metformin ED_50_ dose + curcumin ED_50_ dose). All treatments were suspended in 0.5% CMC and given orally at 10 mL/kg. The number of animals for each experimental group was calculated using G*Power 3.1.9.6 software at 5% type I error (α = 0.05), 80% statistical power (1 − β = 0.8), and 0.5 effect size.

### Formalin test in mice

Before the nociceptive induction, mice were orally administered different treatments (8 mice per group) and placed in acrylic chambers for 1 h for acclimatization to the experimental setup. Behind each acrylic chamber, two mirrors were placed at a 45° angle to facilitate the behavioral observations. Then each mouse received 10 µL of 5% (v/v) formalin in normal saline to the plantar surface of the left hind paw^70^. Immediately after formalin administration, mice were placed back in the acrylic chambers, and pain-like behaviors were recorded for 40 min. Then the recorded videos were analyzed using the Behavioral Observation Research Interactive Software (BORIS)^71^, and the duration of hind paw licking in each 5 min was determined. Hind paw licking in 0–5 min was considered early phase I, wherein 10–40 min period was considered later phase II. The percentage of antinociception in either phase was calculated using the following formula:

3 $$\% {\text{Antinociception }} = { 1}00 \, {-} \, \left[ {\left( {{\text{D}}_{{{\text{treatment}}}} /{\text{D}}_{{{\text{control}}}} } \right) \, \times { 1}00} \right],$$

where D_treatment_ is the duration of hind paw licking for each treated mouse, and D_control_ is the average time of hind paw licking in the vehicle-treated group.

### Analysis of interaction between curcumin and metformin

The interaction between two compounds was determined according to the method described by Tallarida et al*.*^72^. Briefly, dose–response curves for individual treatments and combination were constructed using least-squares linear regression, and the dose resulting in 50% of the effect (ED_50_ ± SEM) was calculated. The theoretical ED_50_ for curcumin-metformin combination was calculated by assuming the occurrence of additive interaction between compounds by using the following equation:

4 $${\text{ED}}_{{{5}0 \, ({\text{add}})}} = {\text{ f }}\left( {{\text{ED}}_{{{5}0 \, ({\text{cur}})}} } \right) \, + \, \left( {{1} - {\text{f}}} \right) \, \left( {{\text{ED}}_{{{5}0 \, ({\text{met}})}} } \right),$$

where ED_50 (add)_ is the theoretical ED_50_; ED_50 (cur)_ and ED_50 (met)_ are experimental ED_50_ of curcumin and metformin, respectively; and f is the fixed ratio of each compound (0.5). Then the theoretical and experimental ED_50_ (ED_50 (mix)_) values for the combination were compared using the t-test. The interaction was interpreted as additive if the ED_50 (add)_ and ED_50 (mix)_ were not significantly different. If the ED_50 (mix)_ was significantly lower or higher than the ED_50 (add),_ the interaction was defined as synergistic or antagonistic, respectively. Moreover, the type of interaction between two compounds was also demonstrated by calculating combination index (CI) and isobologram analysis. The CI was calculated using the following equation:

5 $${\text{CI }} = {\text{ ED}}_{{{5}0({\text{mix}})}} /{\text{ED}}_{{{5}0({\text{add}})}} .$$

The interaction between two compounds was defined as synergistic, additive, and antagonistic if CI < 1, CI = 1, and CI > 1, respectively. Moreover, the type of interaction was also demonstrated by an isobologram as described by Tallarida et al. Briefly, the ED_50_ doses of curcumin and metformin were plotted as axial points in a Cartesian plot, and then the ED_50 (add)_ and ED_50 (mix)_. Then a straight-line connecting ED_50 (met)_ and ED_50 (cur)_ was plotted (additive line). The type of interaction was determined by the location of the ED_50 (mix)_ relative to the additive line.

### Network pharmacology analysis

The SMILES and SDF formats of curcumin and metformin were obtained from PubChem (https://pubchem.ncbi.nlm.nih.gov) and further used to investigate their respective potential targets in several databases, including Swiss Target Prediction (http://www.swisstargetprediction.ch/), Pharmapper (PharmMapper (lilab-ecust.cn)^73^, and Similarity ensemble approach (SEA Search Server (bkslab.org)^74^. These databases can predict the targets of curcumin and metformin at the cellular and mechanistic levels.

Furthermore, inflammatory pain-related targets were used to identify the potential targets in inflammatory pain. Since rheumatoid arthritis is the most common prevalence of inflammatory pain, the keyword of “rheumatoid arthritis” was applied for finding related target genes in several databases, including the DisGeNET database (Fit score ≥ 0.1) (https://www.disgenet.org/), OMIM^®^ database (https://www.omim.org/), GeneCards (Score ≥ 10) (https://www.genecards.org/). *Homo sapiens* was selected as the target species. The target genes obtained from all databases were further unified and standardized using the UniProt database (https://www.uniprot.org/), and the duplicate targets were then removed.

The intersection between the target genes of curcumin, metformin, and curcumin and metformin combination and inflammatory pain-related targets was assessed using Venny 2.1 (https://bioinfogp.cnb.csic.es/tools/venny/). The intersection of genes of curcumin-metformin combination and genes of target diseases were further used as a potential target of curcumin-metformin combination on inflammatory pain. The protein–protein interaction was performed and constructed using the STRING database and exported into Cytoscape software (3.9.1) for further analysis. The cytoHubba v.0.1 plugin of Cytoscape was then used to determine the top 10 genes having a high degree of interactions. To illustrate the potential mechanism of action and pathways, enrichment analysis was performed using Gene Ontology (GO) and Kyoto Encyclopedia of Genes and Genomes (KEGG) pathway enrichment analyses. The KEGG analysis was performed as previously described^75^. GO and KEGG pathway enrichment analyses were performed and visualized using the Online tool of bioinformatic data analysis (http://www.bioinformatics.com.cn/).

### Rotarod test in mice

Three days before the test, mice were trained to remain on a rotating rod at a constant speed (18 rpm) for 180 s every day^76^. On the experiment day, mice were tested before drug administration, and mice capable of remaining on the rod for 180 on three successive trials were used for the subsequent experiment. Four independent animal groups (6 mice per group) were examined for motor coordination after oral administration of either vehicle, the highest dose of curcumin, metformin or curcumin-metformin combination. The latency to fall was recorded at 30-, 60-, 90-, 120-, and 240-min post-treatment with the cut-off value of 180 s.

### Exploratory behaviors by LABORAS

The effect of test compounds on the short-term locomotive behaviors was assessed using an automated home-cage monitoring system, LABORAS (Metris, Hoofddorp, Netherlands). LABORAS system picks up vibrations generated by the movements of the rodent and converts those to the behavioral classifications, including climbing, rearing, locomotion, and immobility^77^. The experimental setup was prepared by adding corn cob bedding to each LABORAS cage (22 cm × 16 cm × 14 cm). Then mice were orally administered with the vehicle, the highest dose of curcumin, metformin (300 mg/kg), and their combination (Met 124.4 + Cur 41.4 mg/kg) (8 mice/group). One hour after, mice were placed in the LABORAS cage, and locomotive behavior was recorded for 30 min. The effect of each compound on short-term locomotive behavior was determined separately for each behavioral measure and presented as a cumulative value of both behavioral duration and frequency.

### General behaviors by LABORAS

LABORAS system facilitates the automatic measurement of rodents’ activity in an undisturbed environment for longer durations. Mice were treated with either vehicle or the highest dose of curcumin, metformin, or their combination (10 mice/group), and placed on LABORAS cages supplemented with food and water. Behavioral measurements were started at 18.00 and recorded for 24 h until 18.00 the next day, both in light and dark cycles. Behavioral analysis was subsequently divided into 2 h intervals. After every experiment, the bedding material, food, and water were removed and replaced after cleaning the cages properly with 70% alcohol.

### Statistical analysis

The data analysis was performed using GraphPad Prism 9.1 (GraphPad Software Inc., La Jolla, CA, USA). All the collected data were summarized with counts and percentages for categorical variables. The numerical values were presented as mean ± SEM. The difference between groups was determined by the One-way analysis of variance (ANOVA) followed by the post hoc test. Statistical significance was considered to be achieved when the p-value was < 0.05 (95% confidence level).

### Ethics declarations

All the animal protocols were approved by the Institutional Animal Care and Use Committee (IACUC) of the Faculty of Pharmaceutical Sciences, Chulalongkorn University (Protocol No. 2033004) and carried out in accordance with the recommendations of the IACUC. All the tests were reported in compliance with the ARRIVE guidelines (Animal Research: Reporting of In Vivo Experiments).

## Supplementary Information

Supplementary Information.

## Data Availability

Data will be made available upon request. Contact pasarapa.c@chula.ac.th.
