# Supplementary material for: Curcumin and metformin synergistically modulate peripheral and central immune mechanisms of pain
Source: Sci Rep. 2022 Jun 11;12:9713. doi: 10.1038/s41598-022-13647-7 (PMC9188603; doi:10.1038/s41598-022-13647-7)
Supplement: Supplementary file 1 — Supplementary Information. [file 41598_2022_13647_MOESM1_ESM.docx]

**Curcumin and metformin synergistically modulate peripheral and central immune mechanisms of pain**

Peththa Wadu Dasuni Wasana^1,2^, Hasriadi^2^, Chawanphat Muangnoi^3^, Opa Vajragupta^4^, Pranee Rojsitthisak^5,6^, Pornchai Rojsitthisak^6,7^ & Pasarapa Towiwat^2,6^*

*^1^Pharmaceutical Sciences and Technology Program, Faculty of Pharmaceutical Sciences, Chulalongkorn University, Bangkok 10330, Thailand*

*^2^Department of Pharmacology and Physiology, Faculty of Pharmaceutical Sciences, Chulalongkorn University, Bangkok 10330, Thailand*

*^3^Institute of Nutrition, Mahidol University, Salaya, Nakhon Pathom 73170, Thailand*

*^4^Molecular Probes for Imaging Research Network, Faculty of Pharmaceutical Sciences, Chulalongkorn University, Bangkok 10330, Thailand*

*^5^Metallurgy and Materials Science Research Institute, Chulalongkorn University, Bangkok 10330, Thailand*

*^6^Center of Excellence in Natural Products for Ageing and Chronic Diseases, Chulalongkorn University, Bangkok 10330, Thailand*

*^7^Department of Food and Pharmaceutical Chemistry, Faculty of Pharmaceutical Sciences, Chulalongkorn University, Bangkok 10330, Thailand*

**The combination of curcumin and metformin decreases expression levels of inflammatory cytokines in LPS-stimulated RAW 264.7 macrophages and BV-2 microglial cells**

The effects of curcumin, metformin, and curcumin-metformin combination on LPS induced proinflammatory cytokine expression (IL-6 and TNF-α) in RAW 264.7 macrophage cells (**Supplementary Table 1**) and BV-2 microglial cells (**Supplementary Table 2**) are given in the table below. As shown in the table curcumin-metformin combination inhibited LPS-induced pro-inflammatory cytokine expression to a greater extent compared to the individual treatments.

| No | Treatment | %Inhibition of IL-6 | | %Inhibition of TNF-α | |
| --- | --- | --- | --- | --- | --- |
|  |  | Mean | SD | Mean | SD |
| 1 | Curcumin 2.5 µM | 18.6 | 4.5 | 21.4 | 1.9 |
| 2 | Curcumin 5 µM | 26.2 | 1.5 | 25.7 | 1.1 |
| 3 | Metformin 0.5 mM | 9.9 | 1.3 | 17.3 | 1.9 |
| 4 | Metformin 1 mM | 11.2 | 3.1 | 20.4 | 0.2 |
| 5 | Combination (2.5 µM CUR + 1 mM Met) | 34.6 | 1.0 | 46.5 | 3.8 |
| 6 | Combination (5 µM CUR + 1 mM Met) | 49.7 | 0.5 | 52.4 | 1.1 |

**Supplementary Table 1.** The combination of curcumin and metformin decreases expression levels of inflammatory cytokines in LPS-stimulated RAW 264.7 macrophages cells.

| No | Treatment | %Inhibition of IL-6 | | %Inhibition of TNF-α | |
| --- | --- | --- | --- | --- | --- |
|  |  | Mean | SD | Mean | SD |
| 1 | Curcumin 5 µM | 37.2 | 7.2 | 22.8 | 7.4 |
| 2 | Curcumin 10 µM | 53.1 | 3.0 | 30.4 | 6.7 |
| 3 | Metformin 0.5 mM | 12.4 | 0.2 | 23.4 | 1.5 |
| 4 | Metformin 1 mM | 21.0 | 3.4 | 29.3 | 1.7 |
| 5 | Combination (5 µM CUR + 1 mM Met) | 70.8 | 10.8 | 29.5 | 6.6 |
| 6 | Combination (10 µM CUR + 1 mM Met) | 87.8 | 3.8 | 64.4 | 9.2 |

**Supplementary Table 2.** The combination of curcumin and metformin decreases expression levels of inflammatory cytokines in LPS-stimulated BV-2 microglial cells.

**The effect of curcumin, metformin and their combination on hind paw licking frequency**

The effect of curcumin, metformin and their combination of hind paw licking frequency is summarized in the figure below (**Supplementary Fig. 1**). The figure indicates intraplantar administration of formalin-induced biphasic hind paw licking behavior. In phase II (10-40 min), the hind paw licking frequencies increased gradually, peaked at 25 min, and declined. Oral administration of curcumin and metformin alone attenuated formalin-induced hind paw licking frequency in a dose-dependent manner. Significant antinociceptive effects in phase I are only observed with the higher doses used for curcumin (100 and 300 mg/kg) and metformin (300 mg/kg). Administration of curcumin-metformin combination produced marked inhibition in hind paw licking frequencies in both phase-I and phase-II of the formalin test. These data are correlated with the hind paw licking duration data (**Fig. 5**).


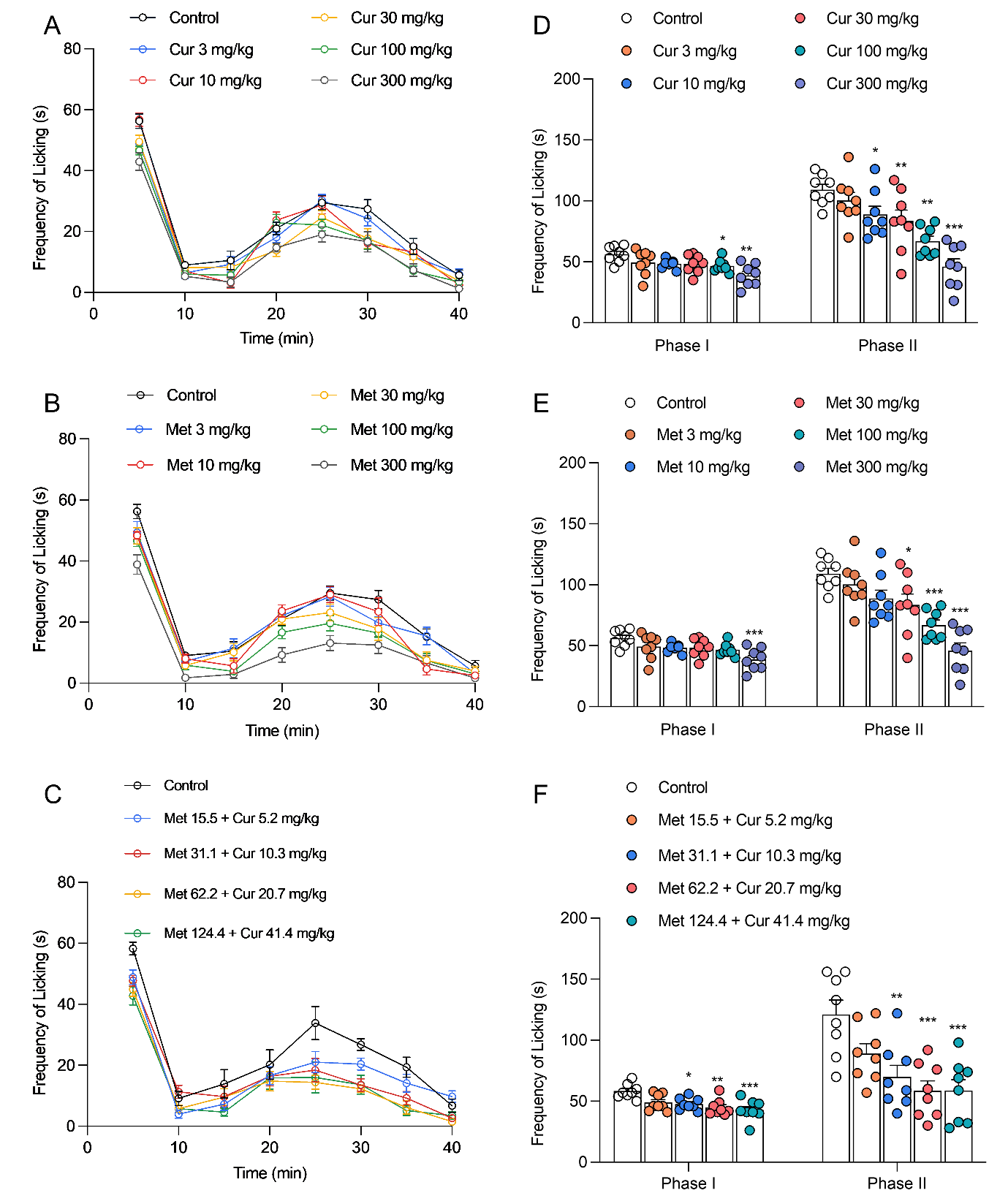


**Supplementary Figure 1. Effect of orally administered curcumin, metformin, and their combination on formalin-induced hind paw licking frequency.** (**A, B, C**) Time course of formalin-induced hind paw licking frequency in mice with oral curcumin (**A**), metformin (**B**), and their combination (**C**). **(D, E, F)** The total frequency of hind paw licking during phases I and II of the pain-like behavioral response with the treatment of curcumin **(E)**, metformin **(F)**, and their combination **(G)**. **p* < 0.05, ***p* < 0.01 and ****p* < 0.001 compared to the vehicle-treated group. One-way ANOVA followed by Bonferroni’s *post hoc* test, n = 8 mice per treatment group.

**Network of the compound-target disease interactions**

The interaction of curcumin and metformin to the target disease-associated genes was visualized using the Cystoscope.

**
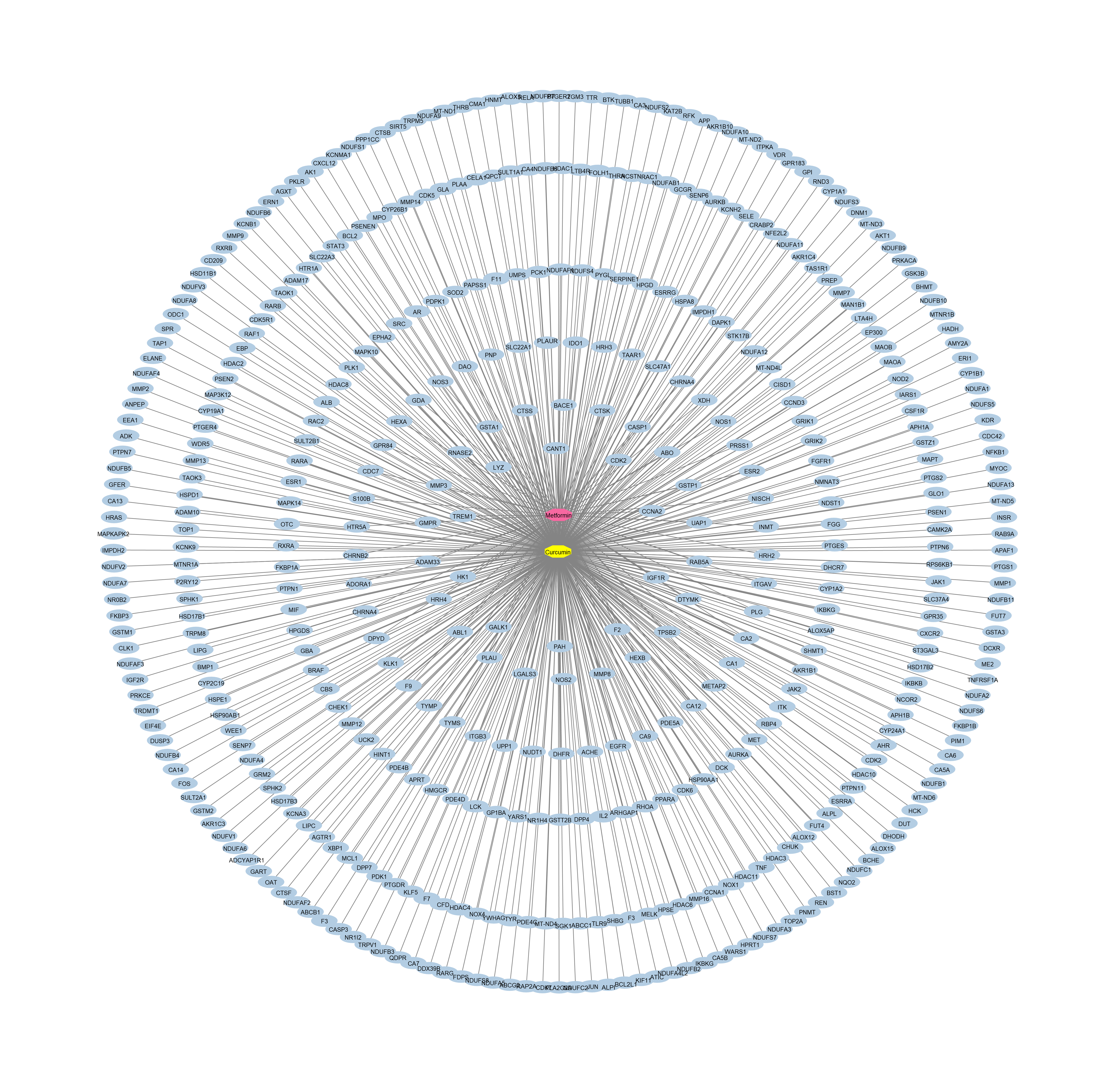
**

**Supplementary Figure 2.** Network interaction of curcumin- and metformin-target disease (rheumatoid arthritis). Yellow, pink, and blue represent curcumin, metformin, and target genes, respectively.
